# Perceptions of Older Men Using a Mobile Health App to Monitor Lower Urinary Tract Symptoms and Tamsulosin Side Effects: Mixed Methods Study

**DOI:** 10.2196/30767

**Published:** 2021-12-24

**Authors:** Elizabeth Y Wang, Benjamin N Breyer, Austin W Lee, Natalie Rios, Akinyemi Oni-Orisan, Michael A Steinman, Ida Sim, Stacey A Kenfield, Scott R Bauer

**Affiliations:** 1 Columbia Vagelos College of Physicians and Surgeons New York, NY United States; 2 University of California San Francisco San Francisco, CA United States

**Keywords:** BPH, mobile health, mHealth, telehealth, telemedicine

## Abstract

**Background:**

Mobile health (mHealth) apps may provide an efficient way for patients with lower urinary tract symptoms (LUTS) to log and communicate symptoms and medication side effects with their clinicians.

**Objective:**

The aim of this study was to explore the perceptions of older men with LUTS after using an mHealth app to track their symptoms and tamsulosin side effects.

**Methods:**

Structured phone interviews were conducted after a 2-week study piloting the daily use of a mobile app to track the severity of patient-selected LUTS and tamsulosin side effects. Quantitative and qualitative data were considered.

**Results:**

All 19 (100%) pilot study participants completed the poststudy interviews. Most of the men (n=13, 68%) reported that the daily questionnaires were the right length, with 32% (n=6) reporting that the questionnaires were too short. Men with more severe symptoms were less likely to report changes in perception of health or changes in self-management; 47% (n=9) of the men reported improved awareness of symptoms and 5% (n=1) adjusted fluid intake based on the questionnaire. All of the men were willing to share app data with their clinicians. Thematic analysis of qualitative data yielded eight themes: (1) orientation (setting up app, format, symptom selection, and side-effect selection), (2) triggers (routine or habit and symptom timing), (3) daily questionnaire (reporting symptoms, reporting side effects, and tailoring), (4) technology literacy, (5) perceptions (awareness, causation or relevance, data quality, convenience, usefulness, and other apps), (6) self-management, (7) clinician engagement (communication and efficiency), and (8) improvement (reference materials, flexibility, language, management recommendations, and optimize clinician engagement).

**Conclusions:**

We assessed the perceptions of men using an mHealth app to monitor and improve management of LUTS and medication side effects. LUTS management may be further optimized by tailoring the mobile app experience to meet patients’ individual needs, such as tracking a greater number of symptoms and integrating the app with clinicians’ visits. mHealth apps are likely a scalable modality to monitor symptoms and improve care of older men with LUTS. Further study is required to determine the best ways to tailor the mobile app and to communicate data to clinicians or incorporate data into the electronical medical record meaningfully.

## Introduction

Lower urinary tract symptoms (LUTS) comprise a complex and heterogenous syndrome, including urinary urgency, urinary frequency, weak urinary stream, hesitancy, straining, incomplete bladder emptying, nocturia, and urinary incontinence [1]. LUTS are chronic and progressive [2], and they affect as many as 72% of men over the age of 40 years [3]. First-line treatment for LUTS due to benign prostatic hyperplasia (BPH) includes medical management with alpha-1-andrenergic receptor blockers, such as tamsulosin. Although some men achieve symptomatic relief with this regimen, many do not, and placebo effects make it difficult for patients and their providers to determine true clinical response [4]. More than 10% of men taking tamsulosin will also suffer from undesirable side effects, such as dizziness, orthostatic hypotension, headache, sexual dysfunction, and rhinitis. In addition to contributing to unnecessary polypharmacy, these potential side effects can be debilitating and a major concern for patients with LUTS, especially in older men or men who achieve minimal or no symptomatic relief from tamsulosin [5]. Thus, successful management of LUTS requires a balance of both benefits and harms from interventions, including tamsulosin, which are highly variable between individuals and may not be accurately communicated or recorded during routine medical encounters. Lowering barriers to improve communication of symptoms and medication side effects may improve understanding and adherence in a significant proportion of men who are incorrectly labeled as refractory to medical LUTS management. In addition, mobile apps may increase patients’ awareness of variability in symptom severity, identify triggers of symptoms or side effects, assist clinicians by quantifying the efficacy and adverse effects of LUTS interventions, and ultimately facilitate shared decision making based on whether a medication is continuing to provide net benefit to the patient.

Mobile apps are increasingly used to monitor symptoms and side effects in a wide range of urologic conditions. For men with LUTS, mobile apps have been successfully used to administer questionnaires [6], increase peak flow rate (ie, with the sound of running water) [7], and guide clinical decision making [8]. Given these findings, a well-designed and thoughtfully implemented mobile app could streamline the way that patients report symptoms and improve characterization of LUTS severity and variability. In addition, mobile health (mHealth) apps may provide an efficient mechanism to generate a repository of patient data that could be used to identify new LUTS phenotypes, define treatment response or harms, and ultimately help clinicians tailor medical management.

To our knowledge, the use of mobile apps to track symptoms and side effects in men with LUTS has not been studied using qualitative methodology. Qualitative studies are uniquely equipped to explore the patient perspective and help ensure that future intervention designs are grounded in the patient experience. Thus, we designed the Placebo-Controlled, Randomized, Patient-Selected Outcomes, N-of-1 Trials (PERSONAL) pilot study, a 2-week intervention to determine the feasibility and acceptability of daily LUTS severity and tamsulosin side-effect assessment through a mobile app among older men with LUTS receiving chronic tamsulosin therapy. Following this study period, we aimed to explore the men’s insights about using an mHealth app to track their symptoms and medication side effects via interviews. Here, we present the findings and implications of the first mixed methods study exploring the experience of men with LUTS after using an mHealth app to track their symptoms and medication side effects.

## Methods

### Study Design

A convenience sample of 19 men was recruited from an academic urology clinic for the PERSONAL pilot study. Recruitment was targeted at men with LUTS who may be unsure if the benefits of tamsulosin outweighed the harms, specifically older men who were both receiving chronic tamsulosin therapy and interested in tracking their daily urinary symptoms and tamsulosin side effects outside of regular clinic visits. Men were eligible if they were taking tamsulosin for LUTS for at least 12 months, received care from a urologist at our institution, and had previously consented to being contacted for research purposes via the electronic medical record. The sample size was based on feasibility of recruitment. Additional inclusion and exclusion criteria are outlined in Multimedia Appendix 1. First, the participants completed a baseline survey describing their LUTS severity and medication side effects; in the same survey, they selected up to three individual symptoms and three tamsulosin side effects to track daily for 2 weeks. Then, the participants underwent an orientation phone call to set up the PERSONAL mobile app; they were guided on how to use the app and had the opportunity to ask questions. The participants received daily questionnaires through the mobile app at a prespecified time of their choice, and the questionnaire data from all participants were then collected in a secure cloud-based database made accessible to the research team; participants could review their own results throughout the study period. Additional study design details were previously published [9], and results pertaining to the PERSONAL pilot study itself will be published in a separate manuscript.

### Interviews

To gain a more nuanced understanding of participants’ experiences using the PERSONAL mobile app following the 2-week data collection period, semistructured feasibility interviews were administered by telephone, ranging from 10 to 30 minutes. The interviewer (EYW) was a medical trainee and research associate with formal qualitative research training, who had no interactions with the participants prior to the interviews. The interview guide is available in Multimedia Appendix 2. Responses were recorded in REDCap (Research Electronic Data Capture) and field notes were made afterward. Due to the relatively structured nature of interview questions, qualitative responses were comprehensively transcribed in REDCap during the phone interviews, but were not audio-recorded. EYW manually coded these open-ended responses using a data-driven approach; codes were iteratively refined into themes. EYW reviewed codes and themes intermittently with coauthors SAK and SRB.

### Statistical Approach

Continuous variables were reported using median and IQR, and categorical data were summarized using frequencies. Thematic analysis of open-ended questions yielded 22 codes, which were refined into eight themes. EYW completed initial coding; codes and themes were finalized in discussion with coauthors SRB and SAK. Qualitative data were integrated relative to quantitative responses [10]. The study is reported in concordance with the Consolidated Criteria for Reporting Qualitative Research (COREQ) checklist [11].

## Results

All 19 participants who participated in the 2-week PERSONAL pilot study completed poststudy interviews; their demographics and self-selected symptoms and side effects are reported in Table 1. All participants found the setup process easy via phone orientation; the majority of participants (n=15, 79%) reported no issues at all, and 4 participants (21%) mentioned minor initial issues, which were easy to resolve (device incompatibility, asked partner to help download app, etc). Some participants suggested an online video or a written document in addition to the phone orientation. Stated reasons for wanting a written document included lower English proficiency and to have materials for future reference.

About two-thirds of participants felt that the questionnaires were just the right length, and about one-third felt that they were too short; the latter participants reported concerns about adequate detail or the appropriate gradations used in the questions and answer choices, commenting that those with milder symptoms might prefer additional gradations on the lower end of the spectrum. Some participants reported frustration about the side-effects questions, as they had difficulty parsing out which side effects to specifically attribute to tamsulosin. A few men were willing to log answers for seven to eight symptoms and side effects rather than the recommended limit of three. About two-thirds of participants set app notifications for mornings, which they found particularly useful for reporting nocturnal symptoms, such as nocturia.

Some participants reported that they did not receive daily notifications via the mobile app due to technical difficulties, but the large majority of participants completed the questionnaire by “habit” at the same time every day—no participants logged responses purely as an immediate response to bothersome symptoms. Out of 19 participants, 9 (47%) felt that this app changed their perception of their health or LUTS management; 8 of these 9 patients (89%) reported increased awareness of symptoms and 1 participant (11%) was able to adjust his fluid intake to improve LUTS. All participants would be willing to share data with their clinician to complement their usual care.

Almost half (8/19, 42%) of the participants reported prior use of other health apps or devices. When prompted to compare those experiences with the study app, most agreed that this app had a simpler interface than others they had encountered, though 1 participant (5%) noted that the app may still be inaccessible in populations with lower English proficiency.

**Table 1:** Participant demographics and responses to structured interviews.

Characteristic		Value (N=19)
Age (years), median (IQR)		70 (62-75)
**Race, n (%)**		
	White	13 (68)
	Black	0 (0)
	Asian	3 (16)
	Other	3 (16)
**Ethnicity, n (%)**		
	Not Hispanic, Latino, or Spanish	16 (84)
	Mexican, Mexican American, or Chicano	1 (5)
	Puerto Rican	1 (5)
	Another Hispanic, Latino, or Spanish origin	1(5)
**Preferred orientation format, n (%)**		
	Online video	10 (53)
	On-demand phone support	6 (32)
	In-person demonstration	4 (21)
	Phone orientation	3 (16)
**Length of daily questionnaire, n (%)**		
	Took more time than it should	0 (0)
	About right	13 (68)
	Took less time than it should	6 (32)
**Time of day questionnaire usually completed, n (%)**		
	Morning	13 (68)
	Afternoon	1 (5)
	Evening	4 (21)
	Sporadic	1 (5)
**Frequency of app usage without alert or notification, n (%)**		
	Never	6 (32)
	A few times per week	11(58)
	More than half of the days per week	1 (5)
	Once per day	2 (11)
	2-3 times per day	0 (0)
	More than 3 times per day	0 (0)
Usefulness of urinary symptom assessment^a^, median (IQR)		4 (4-4)
Usefulness of medication side-effect assessment^a^, median (IQR)		4 (2-5)
Reported change in urinary symptom management due to the use of the PERSONAL^b^ app (during the 2-week study), n (%)		9 (47)
Would allow clinician to see PERSONAL app data, n (%)		19 (100)
Reported use of other health apps or devices, n (%)		8 (42)
**Other health apps or devices, n (%)**		
	Fitbit	4 (21)
	Other^c^	6 (32)

^a^On a scale of 1 to 5, where 1 is “not at all useful” and 5 is “very useful.”

^b^PERSONAL: Placebo-Controlled, Randomized, Patient-Selected Outcomes, N-of-1 Trials.

^c^Other health apps or devices include Fitbit-like device, Apple Watch, MyFitnessPal, Brainscape, and the iPhone heart rate monitor.

**Figure 1:**
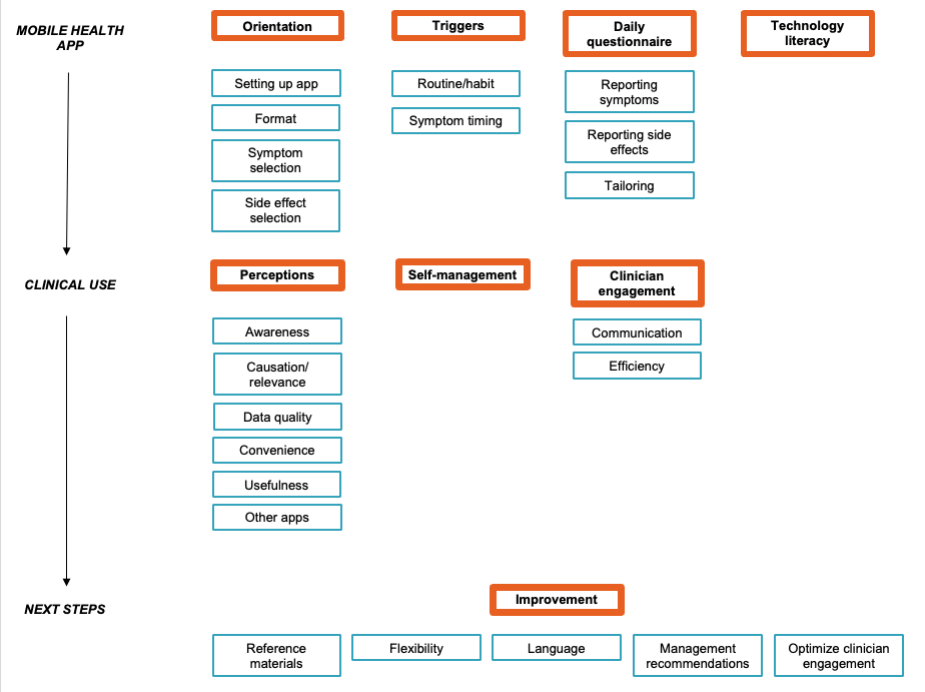
Codes and themes generated from the qualitative analysis. Orange boxes represent themes and blue boxes represent codes.

The qualitative analysis generated eight themes (ie, orientation, triggers, daily questionnaire, technology literacy, perceptions, self-management, clinician engagement, and improvement) and 22 codes organized into three categories (ie, mHealth app, clinical use, and next steps) whose relationships are illustrated in Figure 1. Definitions for themes and select quotations organized by code are included in Table 2. For example, under the theme “perceptions” and code “usefulness,” one participant said, “I learned some things that I didn’t know about my condition before; I was surprised about what I learned about my symptoms—'cause then I can talk to my doctor about it.” Under the theme “clinician engagement” and code “efficiency,” another participant said, “I think it would help that we would be looking at this issue a little more closely as opposed to a casual conversation during the visit. I think the doctor would be able to monitor the frequency of symptoms I’m having either throughout the night or the day and we can decide if the dosage needs to be changed.” Additional quotations are presented in Multimedia Appendix 3.

**Table 2:** Selected participant quotations organized by themes and codes.

Themes and codes			Quotations
**Mobile health app**			
	**Orientation** **: participants discussed the orientation process, from the assistance they received while setting up the app to the symptom and side-effect selection**		
		Setting up app	“Fairly easy. Good to have someone on the other line to take me through it step by step.” “Instructions were very straightforward, and the graphics were easy to read.”
		Format	“The way it was done in the study was good: email and phone orientation.” “Would prefer written documentation in case there were issues.” “Send link by email and I can follow instructions—I don’t need any human contact.”
		Symptom selection	“Disappointed that it was limited to three, would have done up to seven to eight.” “I have more symptoms than the survey asked about—the top three weren’t enough for me.” “The more items I was keeping track of, the more I was attuned to my symptoms, so more questions would be good.”
		Side-effect selection	“Would have preferred to track more side effects. Initially thought some of these were just symptoms, not side effects, like runny nose. I also have allergies, but definitely could have been tamsulosin.”
	**Triggers: participants discussed their experiences and preferences regarding reminders and notifications from the mobile app**		
		Routine or habit	“Never received notifications, I went in and answered by habit.” “Just did the questions before bed, whether I got the notification or not yet that day. I was getting daily notifications.”
		Symptom timing	“Sometimes I would remember to answer questions in the morning before the notification—my symptoms were mostly during the night, so I remembered to log in the morning.”
	**Daily questionnaire** **: participants discussed their experiences completing daily questionnaires within the mobile app**		
		Reporting symptoms	“Asked about symptoms that weren’t applicable and did not ask about leakage.” “Maybe some questions seemed like they would be too simple for others but were good for me.”
		Reporting side effects	“Impossible to answer—whatever side effects could be caused by other things. All of that was kind of irrelevant—couldn’t relate anything to Flomax.” “Hard to distinguish which symptoms are from what.”
		Tailoring	“Frustrated because some issues were not questioned in the survey. Not sure if I’m having them because of meds or not. The survey was simplistic, basic. Would have preferred more depth—and to be able to note the thoughts before this interview.” “Some questions didn’t apply.”
	**Technology literacy: participants discussed their ability to engage with technology**		
		N/A^a^	“It’s very easy for everybody, even people like me, old and not very good with technology.”
**Clinical use**			
	**Perceptions** **: participants discussed their perceptions as a result of using the mobile app**		
		Awareness	“Brought more awareness to the issue; and now I think about it more than before. I kind of wonder how much it affects my sleep and it brings the awareness to the forefront.” “Became more attuned to your symptoms, more attentive—had never previously paid attention.” “Made me more aware of the topic.” “Made me more conscious of [symptoms]. Haven’t seen a doctor in a while, keeps the issues fresh on my mind so I can track them.”
		Causation or relevance	“No relationship between what Flomax does. The app tells you what you’re feeling but doesn’t say why you feel that way.” “I didn’t know if I was getting side effects, and I didn’t know if they were related to the meds or just overall bladder problems; no baseline to compare to.”^b^ “What if you take eight meds? This is just one medication. Lots of meds have the same side effects, ie, headaches, dizziness.”
		Data quality	“The questions were limiting. The number of options for getting up at night were recorded as a range of numbers rather than a precise number. Trying to track things means you want to be more specific.” “[Would prefer] having more longitudinal data—duration of symptoms.” “Do you have this problem 0 times, 1-2, et cetera, well geez I’m more like 0-1, but there’s no way to put 0-1. Having a selection process that takes in the range of options, I would suspect most people the frequency is variable from day to day. So, if you have 0-2, that certainly includes 0-1 and 1-2, and I would think that frequency question should be taken into account, maybe the lower levels more inclusively, rather than feeling like there’s a gap or uncertainty in the choice.”
		Convenience	“Would have loved to continue this study, if possible, I could always record on paper but it’s not as convenient. With the app, it’s just clicking buttons.” “It was short enough to stick to every day.”
		Usefulness	“I learned some things that I didn’t know about my condition before; I was surprised about what I learned about my symptoms—'cause then I can talk to my doctor about it.” “Same answers every day. It’s a one-way survey, not a dialogue—not sure if the answers are good enough to change management.” “Nice to have a tool to help with aging issues.”
		Other apps	“The PERSONAL^c^ app is more user friendly, and you only have to provide simple responses.” “This app was very basic, not even an app, just a questionnaire. An app tries to change behavior—there was nothing in the app that seems to have any influence on behavior.”
	**Self-management:** **participants discuss using the questionnaires to guide self-management**		
		N/A	“More awareness of symptoms—the morning timing is very appropriate for answering the questions. So maybe that day based on my doctor’s recommendations I will change my diet, for example, citrus/salt changes. I analyze why certain nights are worse than others.”
		N/A	“I’m going to talk to my doctor and tell him that I did this and tell him what I learned.”
	**Clinician engagement: participants discuss their preferences for clinician engagement**		
		Communication	“I would like my doctor to have every scintilla of data they can have.” “It made me more aware of leakage, because of incomplete emptying, and I want to share this with her.”
		Efficiency	“It would help: the more that you can do electronically to help your doctor, the less time they have to spend on the office visits, especially given how busy they are. That would be helpful in monitoring.” “I think it would help that we would be looking at this issue a little more closely as opposed to a casual conversation during the visit. I think the doctor would be able to monitor the frequency of symptoms I’m having either throughout the night or the day and we can decide if the dosage needs to be changed.”
**Next steps**			
	**Improvement:** **participants discuss potential areas of improvement for the mobile app and its integration into clinical care**		
		Reference materials	“Maybe save [the symptom list] for participants so they can reference which were symptoms versus side effects.”
		Flexibility	“More open ended, the questions from the app were overly simple.” “Wanted afternoon/evening reminders, but was getting them in the morning, but I was reflecting on the day before rather than for the next 24 hours and that based on the person who set it up and I thought we could change it from AM to PM.”
		Language	“Based on experience as a nurse in Oakland where 90% of patients were Spanish speaking, many underserved patients over the age of 60 would not be able to benefit from this app as it currently stands.” “Because English isn’t first my language, something to read would be best so I can look it up on Google Translate.”
		Management recommendations	“There were no directions on how to manage one’s symptoms based on the answers.” “Maybe add questions in the app about how many cups of water to better help with management; could have scores/goals incorporated in the app to keep you on track, like Fitbit.”
		Optimize clinician engagement	“Yes—of note, I had a video appointment with my urologist during the 2 weeks, and told him about the study. My urologist’s reaction was ‘zero’ because the urologist didn’t know what it was.”

^a^N/A: not applicable; there were no codes under this theme.

^b^N-of-1 is supposed to help establish a baseline to help reduce this type of confusion.

^c^PERSONAL: Placebo-Controlled, Randomized, Patient-Selected Outcomes, N-of-1 Trials.

## Discussion

In sum, mHealth apps may play an important role in the chronic management of LUTS. In this study, the key themes that emerged from our qualitative data were (1) orientation, (2) triggers, (3) daily questionnaire, (4) technological literacy, (5) perceptions, (6) self-management, (7) clinician engagement, and (8) improvement. Consistent with prior literature [12,13], our participants had adequate technology literacy to use this app on a daily basis. The questionnaires were short enough for daily adherence, though a subset of participants with more severe or poorly controlled symptoms believed the questionnaires were not encompassing enough. These participants either felt that the app’s closed-ended question format impeded accurate reporting, that they wanted to track symptoms that were not selected, or they wanted to track symptoms in addition to the ones selected. Given the desire for increased flexibility in communication, certain patients may benefit from a free-text or messaging option. Some patients taking multiple medications expressed frustration at not being able to differentiate which side effects were attributable to tamsulosin, further supporting the need for individualized tracking and potentially individual crossover trials (eg, N-of-1 studies). Patients with prior experience logging health information on a daily basis mentioned that mobile apps are a great way to store data and communicate important information with their clinicians, especially compared with recording on paper. Participants with more severe symptoms seemed less empowered to use their daily logs to change management, potentially because even their clinic visits with their clinicians failed to yield symptomatic improvement, among other reasons.

Most American men and adults over the age of 65 years own a smartphone [14]. Mobile apps are widely used by the general public, and the number of urology-related mobile apps has multiplied in recent decades [15]. These apps support patients with a wide range of urologic conditions, but their usage is unstandardized and unregulated [16]; in addition, mobile app usage remains inconsistently integrated with routine clinical care [17].

Studies support the need for expert involvement in mobile app development, dissemination, and regulation [15,16,18,19]. In our study, patients were unanimously willing to share this health data with their clinicians with the goal of optimizing their urologic care. Likewise, in a British study, urologists reported considerable interest in incorporating various mobile apps into their urologic practices [20]. Leveraging the doctor-patient relationship in the early phases of using the PERSONAL app would likely mitigate both patient confusion regarding which symptoms are medication side effects versus isolated symptoms, as well as clinician confusion when their patients ask to discuss data from a mobile app they are using to track their symptoms. Becoming more aware of symptoms via a mobile app may also help patients hone their questions when visiting their physician. Ultimately, mobile apps may become an important part of LUTS management, such as motivating patients and providers to stop chronic tamsulosin therapy if it is no longer helpful or causing nonspecific yet bothersome side effects. Tracking symptoms regularly using an mHealth app could also potentially help identify low-frequency, but serious, events associated with tamsulosin use, such as falls [21].

This study offers insights into the benefits and patient concerns associated with tracking symptoms via a mobile app among older men with benign urologic conditions, but there are some limitations. Traditionally, qualitative interviews are audio-recorded unstructured or semistructured interviews. Given the focus of the study, the homogeneous population, and the strong convergence of responses around a few common themes, we likely reached data saturation among 19 participants [22]. We solicited a wide range of responses, but less structured interviews in more diverse patient populations are warranted in future studies; additional qualitative studies involving clinical providers could inform the best ways to incorporate the use of mobile apps into clinical care. While qualitative interviews allow study participants to discuss their experiences in more depth, some participants may have felt less candid than they would answering an online form.

Despite the limitations, this is the first mixed methods study to examine the use of a mobile app to track symptoms in older men with LUTS. Importantly, these results suggest that mobile apps designed to improve symptom awareness and management may be adapted to benefit older men with LUTS due to BPH. As the use of mobile apps becomes increasingly popular, their usage in the health care setting will require further optimization. Tailoring to individual patients’ health and technology literacy levels, language proficiency, and symptom severity will be critical to maximizing the efficacy of these digital interventions. Incorporating the use of apps into clinical practice may play another important role, pending future study.

## Appendix

Multimedia Appendix 1 Study inclusion and exclusion criteria.

Multimedia Appendix 2 Interview guide.

Multimedia Appendix 3 Participant feedback.

## Abbreviations

BPH: benign prostatic hyperplasia
COREQ: Consolidated Criteria for Reporting Qualitative Research
LUTS: lower urinary tract symptoms
mHealth: mobile health
PERSONAL: Placebo-Controlled, Randomized, Patient-Selected Outcomes, N-of-1 Trials
REDCap: Research Electronic Data Capture
