# Supplementary material for: Perceptions of Older Men Using a Mobile Health App to Monitor Lower Urinary Tract Symptoms and Tamsulosin Side Effects: Mixed Methods Study
Source: JMIR Hum Factors. 2021 Dec 24;8(4):e30767. doi: 10.2196/30767 (PMC8742207; doi:10.2196/30767)
Supplement: Multimedia Appendix 1 [file humanfactors_v8i4e30767_app1.docx]

Multimedia appendix 1. Study inclusion and exclusion criteria

| **Inclusion Criteria** |
| --- |
| 1. Male patient evaluated by a urologist at the University of California, San Francisco 2. iPhone smartphone or iOS tablet with an active data plan and/or connected to a home WiFi network 3. Age ³55 years 4. Diagnosis code for BPH or other micturition problem based on ICD-10 (ICD10 N40, R35.0-1, R39.11-12, R39-15-16, R39198) 5. Taking tamsulosin daily for ³12 months 6. Downloaded an app from the App Store within the past year 7. Ability to speak and read in English 8. Willing to install the PERSONAL mobile application on their iOS device |
| **Exclusion Criteria** |
| 1. History of acute urinary retention, recurrent urinary tract infections, nephrolithiasis, obstructive kidney disease, urethral stent, or intermittent catherization 2. Active cancer treatment or medical condition that would limit the patient’s life expectancy to <6 months per chart review 3. History of dementia, bipolar disorder, schizophrenia, active suicidality, active substance use disorder, Parkinson’s disease, multiple sclerosis, or prostate cancer per chart review 4. Current participation in another mobile health app-based clinical study 5. Planning to relocate from study area within 6 months 6. Impaired vision that limits the use of mobile health apps 7. Have a health proxy |

Abbreviations:

UCSF- University of California, San Francisco

ICD- International Classification of Diseases
