# Supplementary material for: Perceptions of Older Men Using a Mobile Health App to Monitor Lower Urinary Tract Symptoms and Tamsulosin Side Effects: Mixed Methods Study
Source: JMIR Hum Factors. 2021 Dec 24;8(4):e30767. doi: 10.2196/30767 (PMC8742207; doi:10.2196/30767)
Supplement: Multimedia Appendix 2 [file humanfactors_v8i4e30767_app2.docx]

Multimedia appendix 2. Interview guide

Study ID:

Hello, my name is ______. I am assisting Drs. Bauer, Kenfield, and Breyer with the PERSONAL study to learn about your experience using the PERSONAL mobile health application. We are interested in and appreciate your feedback. There are no right or wrong answers. Your responses will be saved on secure server and your identity will be removed from your comments for analysis by the study team. At the end of this interview, you will be emailed a $50 gift for your participation in the PERSONAL study.

Do you consent to participate in this interview? Yes No

**Support:**

What was the easiest part of setting up the PERSONAL app?

What was the most frustrating aspect of setting up the PERSONAL app?

If you were to be "prescribed" this mobile health an online video application at your doctor's office, would you an in-person demonstration prefer: a phone orientation on-demand support over the phone

What else might be helpful?

**Utility:**

How did you feel about how long it took to complete the questionnaires each day on the PERSONAL app?

Took more time than it should

About right

Took less time than it should

When did you find yourself most using the app?

In the morning

In the afternoon

In the evening

Did you ever use the PERSONAL app when there was not an alert/notification?

Yes

No

How often did you look at the PERSONAL app without being prompted by an alert/notification?

Never

A few times per week

More than half of the days per week

Once per day 2-3 times per day

More than 3 times per day

What made you think about checking in with the PERSONAL app?

On a scale of 1 to 5, where 1 is not at all useful and 5 is very useful:

1Not at all useful

5Very useful

Was the urinary symptom assessment useful? Was the medication side effect assessment useful?

What would make the urinary symptom assessment more useful?

What would make the medication side effect assessment more useful?

Would you change anything about the way we asked you to select the specific urinary symptoms to monitor?

Would you change anything about the way we asked you to select the specific medication side effects to monitor?

**Self-Management:**

Did using the PERSONAL application change how you managed your urinary symptoms during the 2-week study?

Yes

No

Can you give some examples of how use of this technology affected how you managed your urinary symptoms?

Do you have ideas about what we can change about the PERSONAL app to make it fit better into your health care management?

Do you have ideas about what we can change about the PERSONAL app to make it more effective to manage your health?

Would you want your doctor to know your PERSONAL app data?

If the data were shared with your doctor, how do you think sharing your PERSONAL app data might affect how you work with your doctor or health care team on your treatment goals?

Has using PERSONAL app changed your perception of your current health or inspired you to change your care regimen in any way?

Have you used other health apps and/or devices?

Yes

No

What were they?

How would you compare your experience with the PERSONAL app to those other health apps or devices?

Thank you very much for all of your responses. We will be happy to share the results of this work with you on request when the study has completed.
