# Supplementary material for: Perceptions of Older Men Using a Mobile Health App to Monitor Lower Urinary Tract Symptoms and Tamsulosin Side Effects: Mixed Methods Study
Source: JMIR Hum Factors. 2021 Dec 24;8(4):e30767. doi: 10.2196/30767 (PMC8742207; doi:10.2196/30767)
Supplement: Multimedia Appendix 3 [file humanfactors_v8i4e30767_app3.docx]

Multimedia appendix 3. Participant feedback

| ORIENTATION |  |
| --- | --- |
| Setting up app | “It was pretty easy!” |
|  | “The UC doctor was helpful so I wanted to be helpful back. “ |
|  | “Just followed the instructions given in the orientation, was not hard at all” |
|  | “Fairly easy. Good to have someone on the other line to take me through it step by step.” |
|  | “Instructions were very straightforward, and the graphics were easy to read” |
| Format | “The way it was done in the study was good: email and phone orientation” |
|  | “Would prefer written documentation in case there were issues” |
|  | “Send link by email and I can follow instructions--I don't need any human contact” |
|  | “Depends on the quality of the [delivery method]" |
| Symptom selection | “Thought it was limiting at first.” |
|  | “Disappointed that it was limited to 3, would have done up to 7-8” |
|  | “I have more symptoms than the survey asked about--the top 3 weren't enough for me” |
| Side effect selection | “Would have preferred to track more side effects. Initially thought some of these were just symptoms, not side effects, like runny nose. I also have allergies, but definitely could have been tamsulosin.” |
|  | “Given how simple the questions were, would have been more willing to address other side effects, could have done 5 side effects” |
|  | “Didn't like being limited to 3” |
| TRIGGERS |  |
| Routine/habit | “Never received notifications, I went in and answered by habit” |
|  | “Just did the questions before bed, whether I got the notification or not yet that day. I was getting daily notifications” |
| Symptom timing | “Sometimes I would remember to answer questions in the morning before the notification--my symptoms were mostly during the night so I remembered to log in the morning.” |
| DAILY QUESTIONNAIRE |  |
| Reporting symptoms | “Nocturia options should have been 1-2 times vs. 3 or more times.” |
|  | “Asked about symptoms that weren't applicable and did not ask about leakage.” |
|  | “Maybe some questions seemed like they would be too simple for others but were good for me.” |
| Reporting side effects | “Impossible to answer--whatever side effects could be caused by other things. All of that was kind of irrelevant--couldn't relate anything to Flomax” |
|  | “Had picked headaches and dizziness but had never associated them with tamsulosin--there was no place to note this” |
|  | “Hard to distinguish which symptoms are from what.” |
| Tailoring | “Frustrated because some issues were not questioned in the survey. Not sure if I’m having them because of meds or not. The survey was simplistic, basic. Would have preferred more depth--and to be able to note the thoughts before this interview.” |
|  | “More depth, to be more tailored. Same 4 questions every morning” |
|  | “Some questions didn't apply.” |
| TECH LITERACY |  |
|  | “It's very easy for everybody, even people like me, old and not very good with technology" |
|  | “PERSONAL is a simple easy to use app” |
| PERCEPTIONS |  |
| Awareness | “Brought more awareness to the issue; and now I think about it more than before. I kind of wonder how much it affects my sleep and it brings the awareness to the forefront” |
|  | “Made me more aware of the topic” |
|  | “Became more attuned to your symptoms, more attentive--had never previously paid attention” |
|  | “Gave me perspective on how to rate symptoms” |
|  | “Made me more conscious of [symptoms]. Haven't seen a doctor in a while, keeps the issues fresh on my mind so I can track them.” |
|  | “It got me thinking about medications and side effects” |
|  | “Maybe this was to help with compliance--that reminds me, there should've been a question about compliance--but compliance wasn't a problem for me” |
| Causation/relevance | “"How does difficulty going to sleep have anything to do with it?" "Since I don't know what the presumed side effects might be, I didn't really know whether difficulty falling asleep was a side effect or not, or increasing urination a side effect or not a side effect…[and questions like:] Did the medication help you fall asleep—no idea if it was the med, if I was taking it every day.” |
|  | “No relationship between what Flomax does. The app tells you what you're feeling but doesn't say why you feel that way” |
|  | “I didn't know if I was getting side effects, and I didn't know if they were related to the meds or just overall bladder problems; no baseline to compare to” (n-of-1 is supposed to help establish a baseline to help reduce this type of confusion) |
| Data quality | “Feels like the clinic visit is a snapshot but doesn't convey the last few months.” |
|  | “The questions were limiting. The number of options for getting up at night were recorded as a range of numbers rather than a precise number. Trying to track things means you want to be more specific." |
|  | “[Would prefer] having more longitudinal data-- duration of symptoms.” |
|  | "Felt like they were looking for a different answer" but how can you see change in 2 weeks? Basically, meds didn't change, so why would answers change?” |
| Convenience | “Would have loved to continue this study if possible, I could always record on paper but it's not as convenient. With the app, it's just clicking buttons.” |
|  | “It was short enough to stick to every day” |
|  | “It was right to the point” |
|  | “It was pretty straightforward” |
| Usefulness | “Has made me more aware so I can help describe symptoms to doctors” |
|  | “Doctor could monitor trends to see how meds are working and if there's a need to change management.” |
|  | "I learned some things that I didn't know about my condition before; I was surprised about what I learned about my symptoms--cause then I can talk to my doctor about it." |
|  | “Same answers every day. It's a one-way survey, not a dialogue--not sure if the answers are good enough to change management.” |
|  | “Nice to have a tool to help with aging issues.” |
| Other apps | “This was more simple and quick.” |
|  | “This app is targeted for BPH and the others are just general exercise. The app requires input of information--may be intended to be used by a doctor. Whereas the others don't need to input and are just for myself to see.” |
|  | “The PERSONAL app is more user friendly and you only have to provide simple responses.” |
|  | “This app was very basic, not even an app, just a questionnaire. An app tries to change behavior--there was nothing in the app that seems to have any influence on behavior." |
|  | “They're all good tools” |
| SELF-MANAGEMENT | “It got me thinking about medications and side effects. But everything is on a timer--many alerts to organize my meds, so normally very consistent. I would never take myself off a certain medication” |
|  | “More awareness of symptoms--the morning timing is very appropriate for answering the questions. So maybe that day based on my doctor's recommendations I will change my diet, for example, citrus/salt changes. I analyze why certain nights are worse than others.” |
|  | “[Sometimes I would] drink one less coffee a day etc.” |
|  | "I'm going to talk to my doctor and tell him that I did this and tell him what I learned" |
|  | “Conscious of how often I was going to the bathroom-- and was then able to lessen the urge.” |
|  | “Helps monitor symptoms on a day to day basis--makes changes based on own daily analysis. This has made me more aware.” |
| CLINICIAN ENGAGEMENT |  |
| Communication | "I would like my doctor to have every scintilla of data they can have" |
|  | “Absolutely, I always keep my doctors informed about everything” |
|  | “[My urologist] had me keep a detailed diary of voiding (self-cath every 3 hours for 6 weeks)--after doing 23 pages of diary I was so relieved to give back this stack of information. The more information he had about my urinary issues, the better he could take care of me.” |
|  | “They might be interested in the drug interactions. The more a doctor knows the better” |
|  | “It made me more aware of leakage, because of incomplete emptying, and I want to share this with her” |
|  | “It would provide more information for care decisions/interventions” |
|  | “Give my doctor all the information you can" |
| Efficiency | “Difficult to connect with doctors quickly…would be nice to have a conversation with the doctor in the tool. For example, if I had a severe migraine today, I could punch it in and the doctor is notified about the migraine and to say something (i.e. prescribe etc). Otherwise I go to urgent care because regular doctors are too busy” |
|  | “It would help: the more that you can do electronically to help your doctor, the less time they have to spend on the office visits, especially given how busy they are. That would be helpful in monitoring” |
|  | “The doctor could call the patient and try to titrate to improve symptoms” |
|  | “Not sure what it would do to help, but no objections. I had a medication dosage increase already and felt it did not help my symptoms” |
|  | “I think it would help that we would be looking at this issue a little more closely as opposed to a casual conversation during the visit. I think the doctor would be able to monitor the frequency of symptoms I'm having either throughout the night or the day and we can decide if the dosage needs to be changed” |
| IMPROVEMENT |  |
| Reference materials | “Maybe save [the symptom list] for participants so they can reference which were symptoms vs. side effects” |
| Flexibility | “More open-ended, the questions from the app were overly simple” |
|  | “What if you take 8 meds? This is just one medication. Lots of meds have the same side effects i.e. headaches, dizziness” CAUSALITY |
|  | “The more items I was keeping track of, the more I was attuned to my symptoms, so more questions would be good.” SYMPTOMS |
|  | “Do you have this problem 0 times, 1-2, et cetera, well geez I'm more like 0-1, but there’s no way to put 0-1. having a selection process that takes in the range of options, I would suspect most people the frequency is variable from day to day. So if you have 0-2, that certainly includes 0-1 and 1-2 and I would think that frequency question should be taken into account maybe the lower levels more inclusively rather than feeling like there's a gap or uncertainty in the choice.” |
|  | “Wanted afternoon/evening reminders but was getting them in the morning, but I was reflecting on the day before rather than for the next 24 hours and that based on the person who set it up and I thought we could change it from am to pm.” |
| Language | “Based on experience as a nurse in Oakland where 90% of patients where Spanish speaking, many underserved patients over the age of 60 would not be able to benefit from this app as it currently stands” |
|  | “Because English isn't first my language, something to read would be best so I can look it up on Google translate” |
| Management recommendations | “There were no directions on how to manage one's symptoms based on the answers” |
|  | “Maybe add questions in the app about how many cups of water to better help with management; could have scores/goals incorporated in the app to keep you on track, like Fitbit” |
| Optimize clinician engagement | “Yes--of note, I had a video appt. with my urologist during the 2 weeks, and told him about the study. My urologist's reaction was ‘zero’ because the urologist didn't know what it was.” |
